# A case with life-threatening uterine bleeding due to postmenopausal uterine arteriovenous malformation

**DOI:** 10.1186/s12905-015-0163-8

**Published:** 2015-02-17

**Authors:** Emi Sato, Kentaro Nakayama, Kohei Nakamura, Masako Ishikawa, Hiroshi Katagiri, Satoru Kyo

**Affiliations:** Department of Obstetrics and Gynecology, Shimane University School of Medicine, Enyacho 89-1, Izumo, Shimane 6938501 Japan

**Keywords:** Postmenopausal woman, Massive vaginal bleeding, Hysterectomy, Uterine arteriovenous malformation, Uterine artery embolization

## Abstract

**Background:**

Uterine arteriovenous malformation is a rare but life-threatening condition that accounts for 1–2% of massive vaginal bleeding. Uterine arteriovenous malformations are less common after menopause. The condition can be diagnosed using Doppler ultrasound, magnetic resonance imaging, computed tomography, and pelvic angiography.

**Case presentation:**

We report a postmenopausal patient with a uterine arteriovenous malformation who underwent emergency hysterectomy for sudden onset of life-threatening uterine bleeding following an initially successful but ultimately failed uterine artery embolization. Interestingly, it was not difficult to ligate and cut the dilated vessels and we were able to safely perform the hysterectomy with little bleeding in the operative field. The hysterectomy was successful, with most of the intraoperative vaginal blood loss due to the ruptured arteriovenous malformation. One year after surgery, the patient has had no vaginal bleeding.

**Conclusion:**

We consider hysterectomy to be a comparatively safe and effective therapeutic option for postmenopausal women who suffered from uterine arteriovenous malformations with life-threatening uterine bleeding.

## Background

Uterine arteriovenous malformations (AVMs) are rare but life-threatening, consisting of an abnormal connection between an artery and a vein [1]. Uterine AVMs usually occur in women of reproductive age; they are very rare after menopause.

We encountered a postmenopausal woman with massive vaginal bleeding from an AVM. Because she had abundant feeding arteries to the uterus, we felt the risk of copious surgical blood loss was a contraindication to immediate hysterectomy. We performed instead a uterine artery embolization (UAE), which was initially successful, but the patient’s condition progressed. She experienced a rupture of her AVM after a second attempted UAE and subsequently underwent emergency hysterectomy.

Some previous reports describe difficulty with UAE cases but still recommend avoiding hysterectomy due to the risk of massive blood loss [1,2]. However, we consider hysterectomy to be an alternative choice for postmenopausal women who suffered from uterine AVMs with life-threatening uterine bleeding. Although the condition is rare, uterine AVM may be suspected in a patient with postmenopausal vaginal bleeding.

## Case presentation

A 60-year-old postmenopausal woman was referred to our hospital with massive vaginal bleeding. Before menopause at age 43, she had a history of 2 cesarean deliveries. She also had a history of hydatidiform mole, 4 years prior to her first viable pregnancy, for which dilatation and curettage was performed and chemotherapy administered. The patient had a medical history of idiopathic portal hypertension and portal thrombosis and a surgical history of esophageal transection and splenectomy. She was on anticoagulation therapy.

Three years prior to the current presentation, she was seen in another prefecture and found to have dilated vessels around the uterus. There was no further follow-up by gynecology. On the day of her current presentation, she went to a local hospital complaining of sudden-onset continuous vaginal bleeding; she was transported to our hospital by helicopter. Speculum examination revealed continuous bleeding from the cervical os. On bimanual examination, the uterus was the size of a man’s fist, soft and spongy, and non-tender. A urine hCG test was negative. She was anemic, with a hemoglobin of 7.6 g/dL, but no tumor markers were elevated (CA 19–9, CEA, CA 125, SCC).

**Figure 1:**
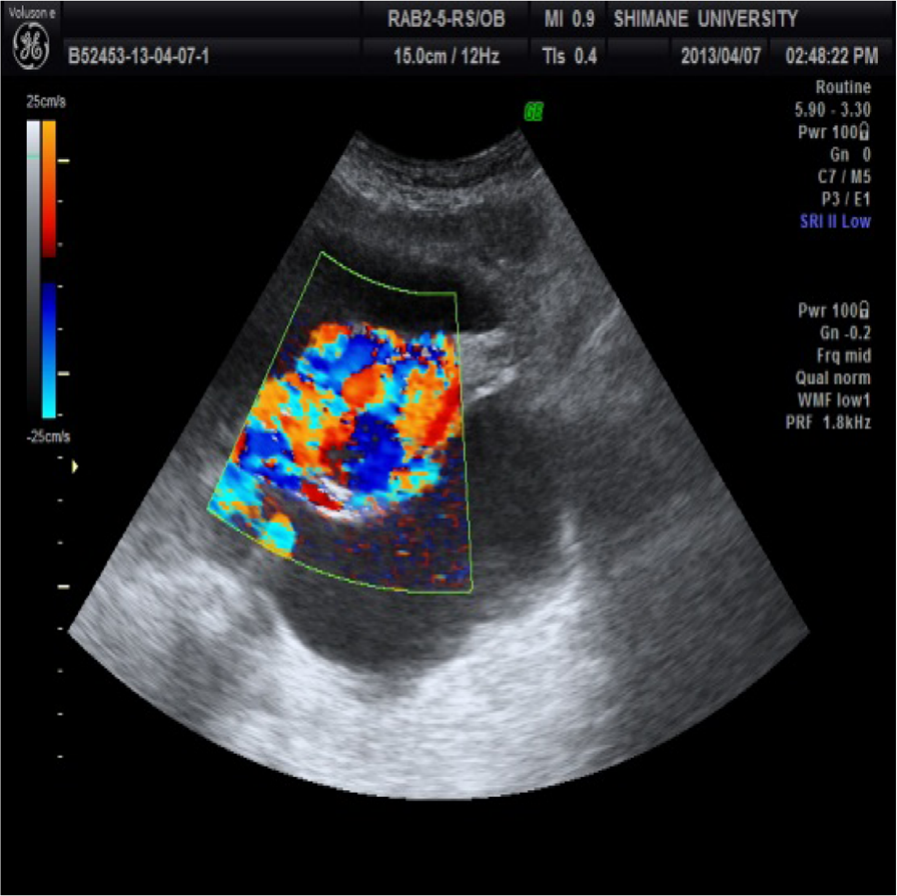
**Color flow Doppler ultrasound shows a mosaic pattern of blood flow within cystic spaces in the uterine body.**

**Figure 2:**
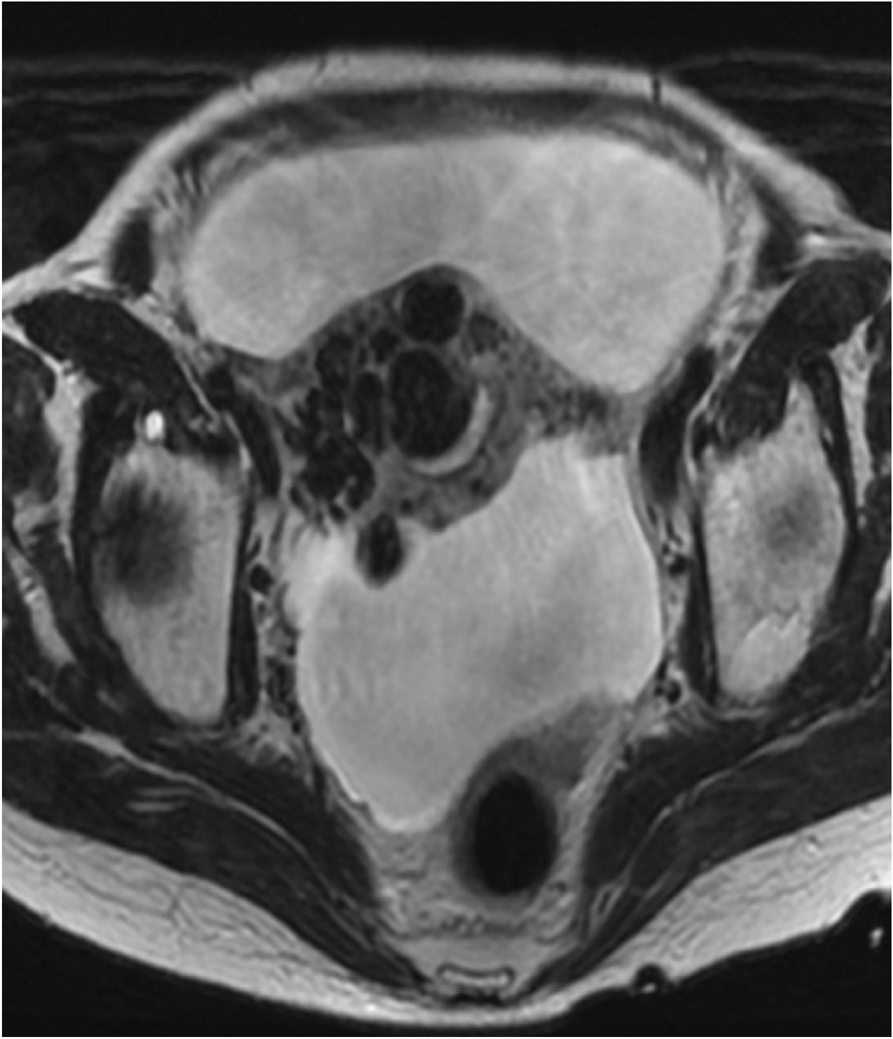
**T2-weighted transverse magnetic resonance imaging (MRI) shows multiple tortuous vessels, especially in the anterior uterine wall.** Ascites caused by the patient’s idiopathic portal hypertension is also present.

**Figure 3:**
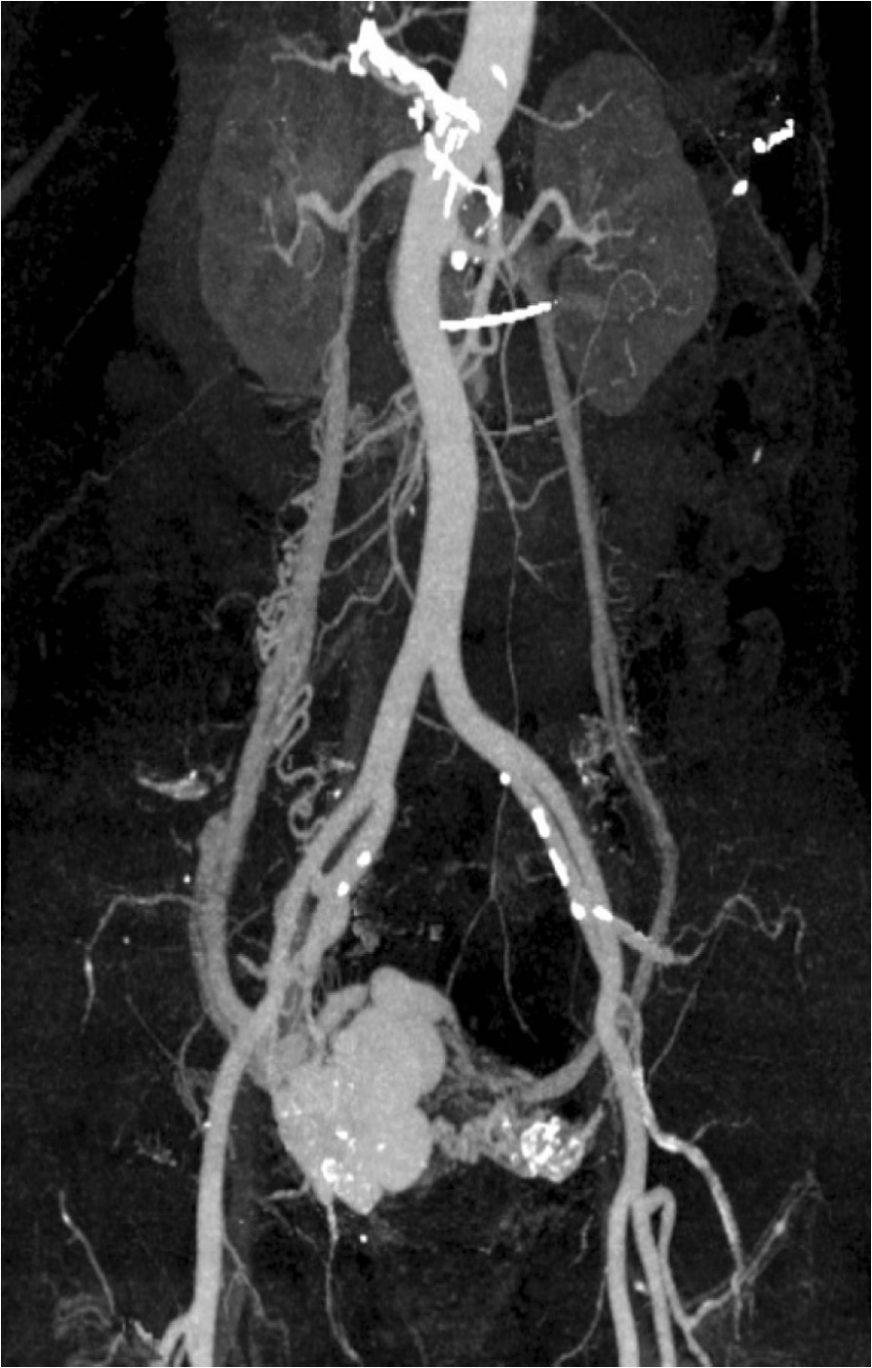
**Computed tomography (CT) angiography suggests multiple expanded vessels around the uterus, including the right uterine artery, right internal iliac artery, right external iliac artery, and left uterine artery.**

Transvaginal sonography showed several 2-cm low-echoic cystic lesions in the anterior uterine body. Color-flow Doppler revealed a mosaic pattern of blood flow within these cystic spaces (Figure 1). Computed tomography (CT) and magnetic resonance imaging (MRI) showed multiple tortuous vessels surrounding the uterus and in the anterior uterine wall (Figure 2). The patient underwent CT angiography which suggested multiple expanded vessels around the uterus (Figure 3). Based on these findings, a uterine AVM was suspected. Given the large vessels supplying the uterus, we anticipated a significant intraoperative blood loss and decided against immediate hysterectomy. We decided to perform UAE.

**Figure 4:**
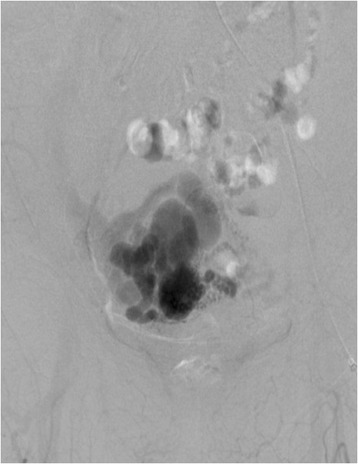
**Pelvic angiography shows a nidus in the right internal iliac artery.**

**Figure 5:**
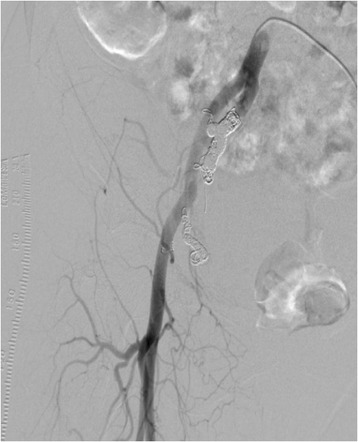
**After embolization of the internal iliac artery using coils and gelatin sponges, there is no significant flow to the AVM.**

**Figure 6:**
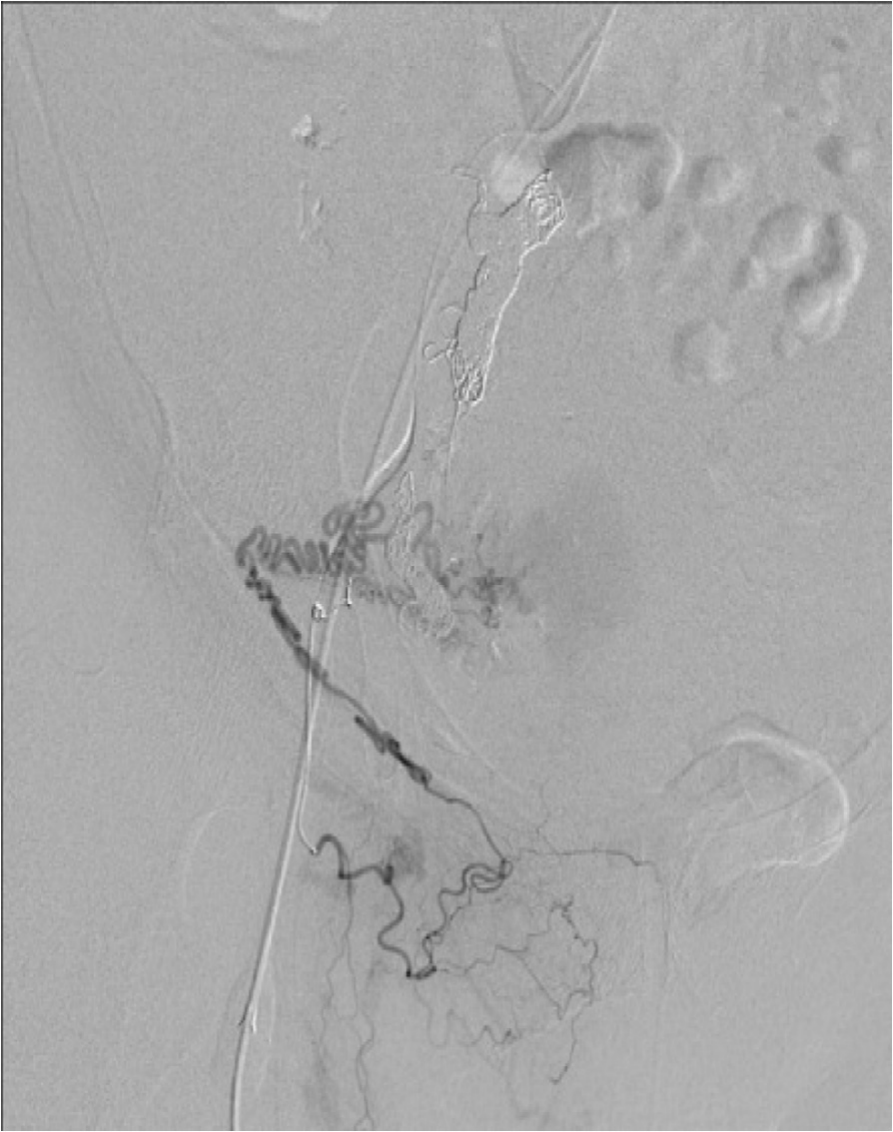
**Eight days after the first embolization, pelvic angiography shows collateral arteries feeding the uterus from the right external iliac artery.**

Pelvic angiography showed that this patient’s AVM was supplied by many arteries, including the right uterine artery, right internal iliac artery, right external iliac artery, and left uterine artery (Figure 4). We performed UAE in the conventional fashion, using coils and gelatin sponges, and found no significant flow to the AVM after the procedure (Figure 5). Eight days after embolization, we repeated pelvic angiography and unfortunately found newly established arteries and collateral vessels feeding the uterus (Figure 6). A second embolization was performed using coils, with n-butyl-2-cyanoacrylate (NBCA) for residual blood flow, but the abundance of feeding and collateral arteries made it difficult to thrombose every vessel. Finally, we concluded that hysterectomy was the better choice and asked the patient for her consent to attempt it as elective surgery. However, 3 days later, a sudden onset of massive vaginal bleeding indicated rupture of the patient’s uterine AVM and we performed an emergency hysterectomy. There were numerous dilated vessels surrounding the uterine corpus. Surprisingly, it was not difficult to ligate and cut the dilated vessels and we were able to perform the hysterectomy with little bleeding in the operative field. The intraoperative blood loss was 2670 mL, but most of this was transvaginal bleeding from the AVM.

**Figure 7:**
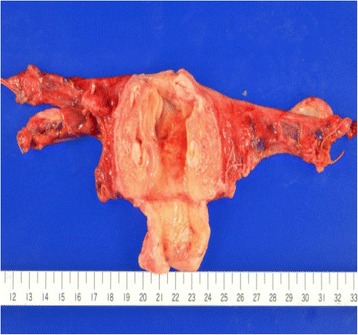
**Macroscopic examination shows dilated vessels; the arteriovenous malformation rupture site cannot be identified.**

**Figure 8:**
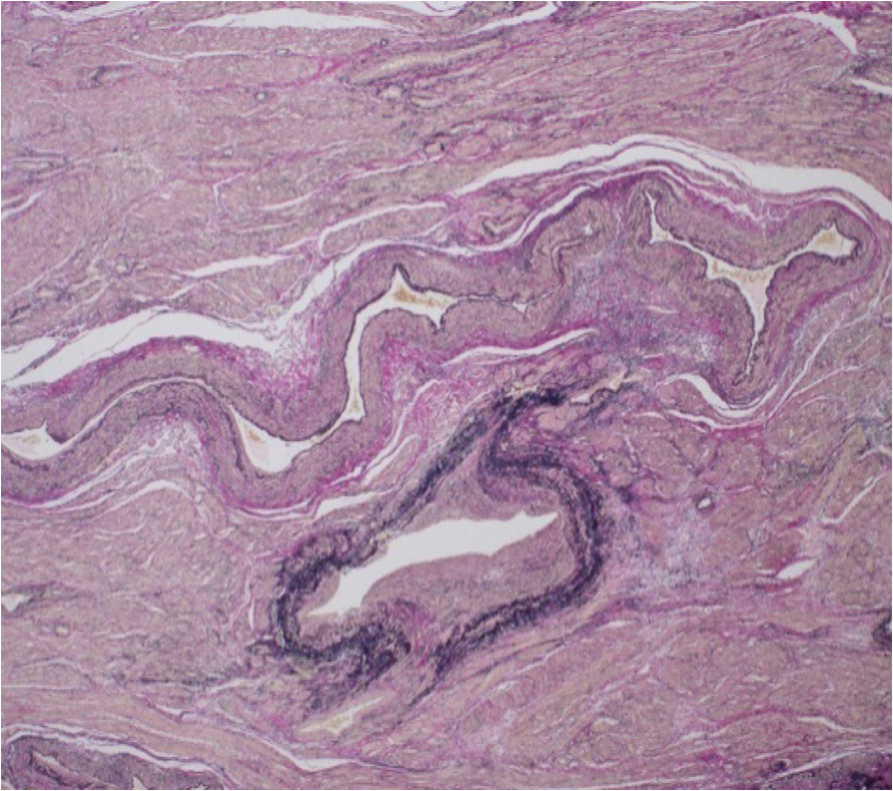
**Microscopic examination shows many dilated thick-walled vessels of varying caliber in the myometrium (hematoxylin-eosin staining; magnification 100×).**

The location of the rupture in the AVM could not be identified, either on macroscopic (Figure 7) or microscopic examination (Figure 8). Microscopy revealed many dilated thick-walled vessels of varying caliber in the myometrium, extending into the endometrium. The final diagnosis of uterine AVM was established. The patient has had no further episodes of atypical vaginal bleeding and no evidence of intraperitoneal hemorrhage 1 year after surgery.

## Discussion

Uterine AVM is a rare cause of abnormal vaginal bleeding, but this bleeding can be life-threatening. A high level of suspicion is required to recognize the presence of uterine AVM in a patient with recurrent, unexplained, massive vaginal bleeding that persists despite medical treatment. Uterine AVM usually occurs during the reproductive years and is most commonly identified when it causes complications during pregnancy [3]. The condition is very rarely seen in postmenopausal women, and only a few reports exist in the literature.

Acquired uterine AVM is more common than congenital uterine AVM and is usually iatrogenic, resulting from prior dilation and curettage, uterine surgery, or therapeutic abortion; the condition can also result from direct uterine trauma, uterine malignancy, and gestational trophoblastic disease [4,5]. Our patient had a history of 2 caesarean deliveries and 1 dilatation and curettage. These findings may be consistent with acquired AVM, she also had idiopathic portal hypertension, which can be associated with uterine AVM.

The treatment options for uterine AVM include surgery and transcatheter UAE, although surgery is usually reserved for those patients for whom UAE is not feasible or is contraindicated [6]. UAE is a well-recognized treatment option for obstetric and gynecologic hemorrhage, and its technical and clinical efficacy has been shown in numerous reports, even in patients with massive bleeding [7]. However, there are some previous reports that describe difficulty with UAE in the presence of AVM [1,2]. Yokomine et al. reported that difficult UAE can result depending on the caliber of the internal iliac artery, the available embolic materials, the amount of blood flow, and the presence of tortuous arteries and abundant feeding arteries [1]. In the present case, we observed the rapid development of collateral vessels and, on the second attempt, were dealing with abundant feeding vessels from not only the internal iliac artery but also the external iliac artery and ovarian artery. We were therefore not able to occlude every possible feeding artery.

## Conclusions

Although some previous authors recommend avoiding hysterectomy, even when UAE proves difficult, because of the risk of massive blood loss [1,2], we were able to proceed with definitive surgery despite the presence of numerous feeding arteries. Although embolization is the most commonly performed first-line treatment for uterine AVM, it is not always successful and subsequent management may be needed [8]. We consider hysterectomy to be a comparatively safe and effective therapeutic option for postmenopausal women who suffered from uterine AVMs with life-threatening uterine bleeding.

## Consent

Written consent was obtained from the patient for publication of this Case report.

## Abbreviations

AVM: Arteriovenous malformation
UAE: Uterine artery embolization
NBCA: N-butyl-2-cyanoacrylate
